# Pyrolysis Kinetics-Driven Resin Optimization for Enhanced Reliability in Ceramic Vat Photopolymerization Manufacturing

**DOI:** 10.3390/ma18174004

**Published:** 2025-08-27

**Authors:** Yun-Zhuo Zhang, Zi-Heng Wang, Wei-Jian Miao, Fan-Bin Wu, Shu-Qi Wang, Jia-Hu Ouyang, Ya-Ming Wang, Yong-Chun Zou

**Affiliations:** School of Materials Science and Engineering, Harbin Institute of Technology, Harbin 150001, China; 21b909010@stu.hit.edu.cn (Y.-Z.Z.); 23s009051@stu.hit.edu.cn (Z.-H.W.); 23s009061@stu.hit.edu.cn (W.-J.M.); 23s109209@stu.hit.edu.cn (F.-B.W.); wangyaming@hit.edu.cn (Y.-M.W.); zouyongchun@hit.edu.cn (Y.-C.Z.)

**Keywords:** pyrolysis kinetics, vat photopolymerization manufacturing, Si_3_N_4_ ceramics, debinding

## Abstract

Thermal debinding represents a critical step that determines the overall success or failure in ceramic vat photopolymerization manufacturing (ceramic VPP), which involves the pyrolysis and subsequent removal of resin (binder). Despite its importance, research into the underlying mechanisms of resin pyrolysis has been largely overlooked. In this study, the multi-distribution activation energy model (M-DAEM) with the pattern-search method and local search algorithms and thermogravimetric data were employed to obtain the kinetic parameters of the resin cross-linked network in printed Si_3_N_4_ green bodies containing monomers with different functionalities, including HEA, HDDA, and PPTTA. High-quality global fitting results were achieved with R^2^ values exceeding 0.9999 across all samples. The kinetics model was further utilized for the numerical analysis of the gas pressure inside the green body during the debinding process. This study indicates that monofunctional monomers can effectively reduce the activation energy of the primary pseudo-components, enabling pyrolysis to occur at a lower temperature and a lower rate, suppressing the peak value of gas pressure, and promoting high-quality debinding of the green body. This study can provide a reference for optimizing the resin formulation in ceramic VPP from the perspective of enhancing debinding performance.

## 1. Introduction

Since the last century, additive manufacturing of ceramic materials has garnered increasing attention, driven by the demand for complex-structured ceramic products in high-tech industries such as aerospace [1,2], biomedicine [3,4], and chemical engineering [5,6]. Among these technologies, ceramic vat photopolymerization manufacturing (ceramic VPP) has emerged as a key research focus due to its extraordinary precision [7,8,9]. The range of ceramic systems applicable to this technology has expanded from easy-to-print materials like SiO_2_ [10,11], Al_2_O_3_ [12], and ZrO_2_ [13] to more challenging ceramics such as Si_3_N_4_ [14], AlN [15], SiC [16], and precursor-derived ceramics [17]. Enhancing the binder system to optimize rheological and curing properties not only improves printing quality but also expands material diversity [18,19]. Nevertheless, irrespective of the specific binders and ceramic fillers used in the slurry, ceramic VPP must address the critical challenge of debinding (commonly referred to as thermal debinding). Given that binders typically constitute 40–70 vol.% of the green body, their removal without damaging the fragile body remains a significant technical hurdle.

Debinding technology has undergone a transformation from simple heating strategies to more complex holding platforms and from oxidative atmospheres to inert atmospheres [20]. Specifically, thermal analysis techniques such as thermogravimetry (TG), differential thermal analysis (DTA) and differential scanning calorimetry (DSC) are employed to identify critical points of weight loss transitions, which enable the establishment of stepwise debinding processes through carefully designed holding platforms. The adoption of inert atmospheres transforms the intense combustion of binders in air into a more controlled and gradual endothermic pyrolysis. All these strategies aim to ensure that gas generation and release perform gradually, thereby minimizing the defect formation during debinding [21]. This is crucial for the manufacturing of large-size/thick-wall ceramic parts due to the longer path for the pyrolysis gas to escape. Gu et al. have fabricated alumina parts with a thickness greater than 10 mm [21]. Marie et al. have achieved the debinding of more challenging centimeter-scale silicon nitride parts. Nevertheless, these empirical debinding processes involve heating for tens to hundreds of hours [22]. Moving forward, enhancing debinding efficiency will require a deeper understanding of the underlying mechanisms, in other words, the kinetics of resin pyrolysis. Regrettably, while current kinetic studies have focused on manufacturing methods such as gel casting [23] and binder jetting [24], limited attention has been devoted to resin pyrolysis in ceramic VPP.

At present, research lacks a pyrolysis kinetics model for various binders applicable to ceramic VPP and methods for predicting the crack risk of green bodies during the debinding process. In this paper, based on the multi-distribution activation energy model (M-DAEM), the debinding behavior of green bodies printed from three different monomer-configured Si_3_N_4_ ceramic slurries was examined. After obtaining thermogravimetric analysis results, the optimized kinetic parameters of the resin were determined through a combination of pattern-search method and local search algorithms. The impact of the functionality of monomers on the pyrolysis characteristics of cross-linked resins was analyzed. The model fitting results exhibited excellent consistency with the experimental data. Subsequently, the kinetics model was incorporated into a numerical model, which accounted for the porosity changes during the debinding and the transport of the pyrolysis gas within the porous medium, aiming to predict the gas pressure variations inside the green body. Finally, Si_3_N_4_ ceramics with high relative density and great mechanical property were successfully fabricated using the optimized resin formulation. These findings can be extended to other ceramic systems and hold implications for the optimization of binders as well as the process improvement of debinding in ceramic VPP, achieving more robust, time-efficient, and energy-efficient ceramic VPP.

## 2. Experiments and Methods

### 2.1. Raw Materials of Si_3_N_4_ Ceramics

Si_3_N_4_ powders (d_50_ = 0.7 μm, purity ≥ 99.9%, Qingdao Cixing New Materials Co., Ltd., Qingdao, China) were used as the main raw materials for the ceramics. Al_2_O_3_ powders (d_50_ = 130 nm, purity ≥ 99.9%, Taimei Chemicals Co., Ltd., Tokyo, Japan) and Y_2_O_3_ powders (d_50_ = 50 nm, purity ≥ 99.99%, Shanghai Naiou Nano Technology Co., Ltd., Shanghai, China) were used as sintering aids. Nano-sized sintering additives can disperse more uniformly in the ceramic matrix, thereby enhancing mechanical properties [25]. The mass ratio of Si_3_N_4_:Al_2_O_3_:Y_2_O_3_ was 90:6:4. Notably, prior to slurry preparation, the Si_3_N_4_ powders were partially oxidized to enhance the curing performance of the slurry and holding for 30 min at 1200 °C in air was determined to be suitable. This process can effectively reduce the absorbance of the powders and mitigate the refractive index mismatch between the powders and resin, thereby improving the overall curing performance [26].

### 2.2. Preparation of Ceramic Slurry

The ceramic slurry used for printing consists of the solid loading component and photosensitive resin. The photosensitive resin comprises monomers, photoinitiator (Omnirad 819, IGM Resins, Waalwijk, The Netherlands), and dispersant (Solsperse 41000, Lubrizol, Wickliffe, OH, USA). These components are mixed at 1200 rpm for 30 min. In this study, all resins contain 1,6-hexanediol diacrylate (HDDA, Covestro, Singapore) due to its excellent dilution capability and versatility. Tetrafunctional ethoxylated pentaerythritol tetraacrylate (PPTTA, Covestro, Singapore) and monofunctional 2-hydroxyethyl acrylate (HEA, Shanghai Macklin Biochemical Co., Ltd., Shanghai, China) are individually or jointly combined with HDDA for investigation. The prepared photosensitive resin was then homogenized with the ceramic powders in a planetary ball mill at 400 rpm for 4 h to make the ceramic slurry, which was degassed subsequently using a vacuum degassing mixer after removing the ZrO_2_ balls to mitigate the oxygen inhibition effect during the photopolymerization process [27]. The specific components of the different slurries are summarized in Table 1.

### 2.3. Preparation of Si_3_N_4_ Ceramics

**Figure 1:**
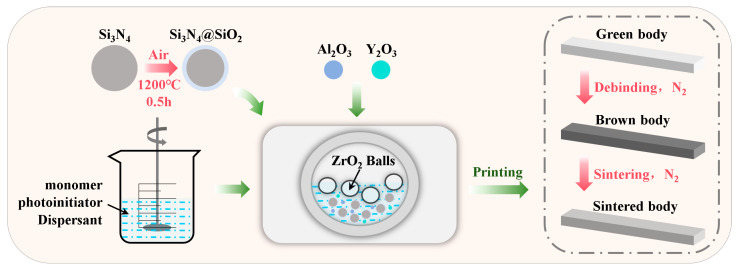
Schematic diagram of the preparation process of Si_3_N_4_ ceramics.

Si_3_N_4_ ceramic green bodies were fabricated using a bottom-up digital light processing printer (Deskcera-P2, Wuhan 3DCeram Technology Co., Ltd., Wuhan, China) with a wavelength of 405 nm. The exposure parameters were optimized based on the curing characteristics of the slurry with a layer thickness of 20 μm. Subsequently, the as-printed green bodies were placed in a tube furnace (VBF-1200X-H8, Hefei Kejing Materials Technology Co., Ltd., Hefei, China) and heated according to a predefined thermal profile for debinding. The samples were then pressureless sintered under N_2_ protection at 1800 °C with a holding time of 120 min using a high-temperature sintering furnace (High-Multi 10000, Fuji Electronic Industrial Co., Ltd., Saitama, Japan). The complete preparation procedure for Si_3_N_4_ ceramics is illustrated in Figure 1.

### 2.4. Characterization

The rheological behavior of the ceramic slurry was tested using a rotational rheometer (HAAKE Viscotester iQ, Thermo Fisher Scientific, Waltham, MA, USA). The phase composition of the Si_3_N_4_ ceramics was analyzed via X-ray diffraction (XRD). The microstructure of the Si_3_N_4_ ceramics was examined using scanning electron microscopy (SEM) (TESCAN MAGNA, Tescan, Brno, Czech Republic). The morphology, microstructure and element distribution of the pre-oxidized powders were characterized with SEM, transmission electron microscopy (TEM) combined with energy-dispersive X-ray spectroscopy (EDS) (Talos F200X G2, Thermo Fisher Scientific, Waltham, MA, USA). The relative density of the ceramics was obtained using the Archimedes method. The sintered ceramic bodies were subjected to a stepwise grinding process using SiC papers with grit sizes ranging from 400 to 2000, followed by fine polishing with diamond spray of 1 μm to 0.5 μm particle size. The processed specimens (3 mm × 4 mm × 36 mm) were utilized for flexural strength testing, which were performed on an electronic universal testing machine (Instron 5569, Instron, Norwood, MA, USA) in three-point bending mode at room temperature and a crosshead speed of 0.5 mm/min with a span of 30 mm, in accordance with ISO 14704:2016 [28].

The pyrolysis kinetics of the resin in green bodies were based on the TG test. Test samples were scraped from the printed green bodies and crushed to particle sizes below 1 mm in order to minimize the temperature gradient. Each sample of 8 mg was uniformly spread in an alumina crucible and introduced into a thermal gravimetric analyzer (TGA/DSC3+, Mettler Toledo, Greifensee, Switzerland) for testing, which was conducted under N_2_ atmosphere at a heating rate of 5 °C/min, with a temperature range of 35–650 °C and a gas flow rate of 50 mL/min.

### 2.5. Pyrolysis Kinetics Model

The pyrolysis of cured resin involves complex reaction mechanisms; therefore, the multi-distributed activation energy model (M-DAEM) is employed to describe it accurately. Typical DAEM assumes that pyrolysis comprises a large number of irreversible, independent, and parallel *n*-order reactions with identical frequency factors, while the M-DAEM consists of several parallel DAEM models [29]. In this study, the cured resin is considered to be composed of three pseudo-components to characterize distinct reaction processes, and the activation energy of each pseudo-component follows a probability density function described by $f_{i}\left(E\right)$, which is widely adopted Gaussian distribution and can be expressed as follows:

(1) $$f_{i}\left(E\right)=\frac{1}{\sigma_{i}\sqrt{2\pi }}exp\left(-\frac{{\left(E-E_{0,i}\right)}^{2}}{2{\sigma_{i}}^{2}}\right)$$

where $E_{0,i}$ is the mean and $\sigma_{i}$ is the standard deviation. Given that the common first-order reaction DAEM can accurately describe the pyrolysis kinetics, the reaction order n is set to 1 [29]. The conversion rate $\alpha_{i}$ of pseudo-component i at temperature T can be expressed as follows [30]:

(2) $$\alpha_{i}=1-\int_{0}^{\infty }exp\left[-\int_{T_{0}}^{T}\frac{A_{i}}{\beta }exp\left(-\frac{E}{RT}\right)dT\right]f_{i}\left(E\right)dE$$

where $T_{0}$ is the initial temperature and $A_{i}$ is the frequency factor of the pseudo-component i. R and β represent the gas constant and the heating rate, respectively. The mass fractions of the three pseudo-components in the cured resin are denoted as $c_{1}$, $c_{2},$ and $c_{3}$, respectively, which satisfy the following condition:

(3) $$\sum_{i=1}^{3}c_{i}=1$$

The calculated normalized mass $m_{Cal}$ of the green body at temperature T can be expressed as follows:

(4) $$m_{Cal}=m_{0}+\sum_{i=1}^{3}c_{i}\;\alpha_{i}\;$$

where $m_{0}$ is the normalized mass of the pyrolysis residue and ceramic powders. The TG data are directly utilized for fitting to avoid systematic errors caused by noise amplification in the derivative thermogravimetric (DTG) data [31]. Numerical solutions containing unknown parameters such as $E_{0,i}$, $\sigma_{i}$, $A_{i}$, and $c_{i}$ can be derived based on the above equations.

The double integral involving E and T in the aforementioned equations will impose a significant computational burden. Therefore, the 4-degree approximation of the Arrhenius function integral is introduced to substantially simplify the calculation [32], as follows:

(5) $$\int_{T_{0}}^{T}exp\left(-\frac{E}{RT}\right)d^{T}\approx \frac{E}{R}\frac{exp(-x)}{x}(\frac{x^{3}+18x^{2}+86x+96}{x^{4}+20x^{3}+120x^{2}+240x+120})$$

(6) $$x=\frac{E}{RT}$$

Numerous studies have demonstrated that during the fitting process, there is a significant kinetic compensation effect, leading to non-uniqueness in the solution [33,34,35]. In other words, different combinations of parameters can yield equally good-fitting results, thereby complicating comparisons. Therefore, in this study, the frequency factors of each pseudo-component are fixed as constants to emphasize variations in activation energy. The specific values are determined through global fitting across all samples and can be referred to similar systems. The former study has shown that the deviation in the simulated weight loss at lower heating rates is not so sensitive to the values of frequency factors [36]. Hence, this method will have great adaptability to the debinding process with slow heating.

The Levenberg–Marquardt algorithm [30] and the Pattern–Search method [37] are commonly employed methods for addressing parameter optimization problems in the DAEM. The former is a gradient-based local search algorithm capable of rapidly identifying local optima but exhibits sensitivity to initial value selection and struggles to locate the global optimum. The latter is a derivative-free global optimization algorithm that performs searches over broad ranges to identify better solutions but may not converge to the absolute optimum. Consequently, in this study, both algorithms are combined: the Pattern–Search method is first utilized for global exploration, followed by the Levenberg–Marquardt algorithm to refine and obtain the final solution. The objective function is defined as follows:

(7) $$O.F.=\sum_{i=1}^{N}{\left(m_{Exp}-m_{Cal}\right)}^{2}$$

Here, N is the number of data points, and $m_{Exp}$ represents the experimentally measured normalized mass of the green body. Furthermore, $R^{2}$ is utilized to assess the fitting quality within any given temperature range [30]:

(8) $$R^{2}=1-\frac{\sum_{i=1}^{N_{r}}{\left(m_{Exp}-m_{Cal}\right)}^{2}}{\sum_{i=1}^{N_{r}}{\left(m_{Exp}-\bar{m_{Exp}}\right)}^{2}}$$

In which, $N_{r}$ and $\bar{m_{Exp}}$ represent the number of data points and the mean value of the experimental data within the specified temperature range, respectively.

### 2.6. Numerical Analysis of Gas Pressure During Debinding

To provide insights into the internal gas pressure evolution within the green body during the debinding process, particularly with respect to the effects of different resin systems, a simplified one-dimensional numerical model in cylindrical coordinates is employed. The model incorporates the M-DAEM of cured resin pyrolysis kinetics, accounts for porosity variations during debinding, and treats the release of pyrolysis gas as seepage flow through a porous medium. For model simplification, a uniform temperature distribution throughout the green body is assumed, which is a reasonable approximation for small-size green bodies. The degree of conversion α of the cured resin at various temperatures can be expressed as follows:

(9) $$\alpha =\sum_{i=1}^{3}c_{i}\;\alpha_{i}$$

Then the volumetric yield of pyrolysis gas can be given as follows:

(10) $$q=\left(1-V_{c}-V_{0}\right)\left(1-\alpha \right)\frac{d\alpha }{dt}$$

In which, $V_{c}$ can be approximately regarded as the volume fraction of ceramic particles. $V_{0}$ is the initial volume fraction of pores, and t is the time. Based on the continuity of gas transport within the green body, the following relationship can be derived [38]:

(11) $$\frac{\partial (\rho_{g}e)}{\partial t}+\frac{1}{r}\frac{\partial (\rho_{g}rv)}{\partial r}=\rho_{p}q$$

Here, $\rho_{g}$ and $\rho_{p}$ are the densities of the pyrolysis gas and the cured resin, respectively. r represents the radial position, and v is the average velocity of the gas flow. Given that the volume change of the green body during the debinding process is negligible, the porosity of the green body can be expressed as:

(12) $$e=V_{0}+\left(1-V_{c}-V_{0}\right)\alpha$$

Gas flow in porous media models adheres to the momentum conservation principle. Furthermore, given the quite low Reynolds number, Darcy’s law can be applied [39]:

(13) $$v=-\frac{K_{g}}{\mu_{g}}\frac{dP}{dr}$$

where $K_{g}$ and $\mu_{g}$ are the permeability and gas viscosity, respectively. P represents the gas pressure of concern. $K_{g}$ can be calculated using the Kozeny–Carman equation [40]:

(14) $$K_{g}=\frac{e^{3}d^{2}}{180{\left(1-e\right)}^{2}}$$

Here, d is the diameter of ceramic particles. Due to the lack of detailed data on the pyrolysis products of the cured resin in ceramic VPP, an accurate value of $\mu_{g}$ is difficult to obtain. Previous studies have indicated that the pyrolysis of most acrylic resins proceeds via a depolymerizing reaction, yielding various low-molecular-weight acrylates [41,42,43]. Accordingly, this study assumes the pyrolysis products to be methyl acrylate, the viscosity of which at temperature T is obtained by fitting a cubic polynomial to the $\mu_{g}$-T curve measured by Gallant [44], as expressed in the following:

(15) $$\mu_{g}=(-10.95996+0.30271T+(-3.20946\times 10^{-5})\;T^{2}+(-3.03030\times 10^{-8})T^{3})\times 10^{-7}$$

The gas pressure adheres to equation of ideal gas:

(16) $$P=\rho_{g}\frac{R}{M_{g}}T$$

where $M_{g}$ and R are the molecular mass of gas and gas constant, respectively. By combining the aforementioned equations, the gas pressure at various temperatures and radial positions can be numerically solved by the implicit Finite Difference Method. The values of the parameters used are shown in Table 2.

## 3. Results and Discussion

### 3.1. Printing Properties of Slurry

**Figure 2:**
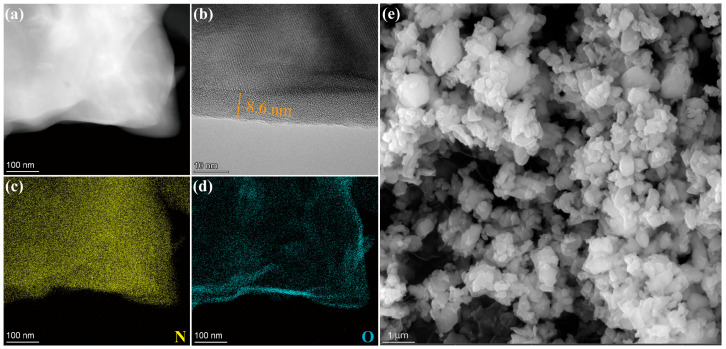
SEM, TEM, and EDS characterization of pre-oxidized Si_3_N_4_ powders: (**a**) HAADF, (**b**) HRTEM, (**c**) N element distribution, (**d**) O element distribution, (**e**) SEM morphology.

The morphology, microstructure, and element distribution of the pre-oxidized Si_3_N_4_ powders were characterized using SEM, TEM, and EDS, as shown in Figure 2. After being treated in air at 1200 °C for 30 min, a noncrystalline layer with a thickness of 8 to 10 nm formed on the surface of the original Si_3_N_4_ particles, as shown in Figure 2b. The energy spectrum results revealed that this amorphous phase was rich in oxygen and deficient in nitrogen, indicating that the Si_3_N_4_ powders had been successfully oxidized to form a Si_3_N_4_@SiO_2_ core–shell structure.

**Figure 3:**
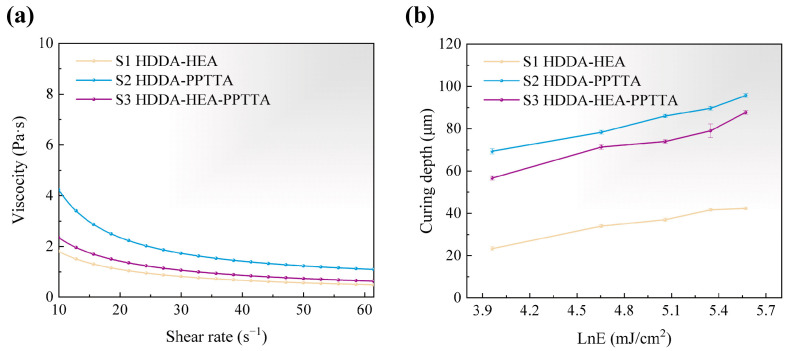
Si_3_N_4_ slurry: (**a**) rheological behaviors, (**b**) curing properties.

Different monomer formulations significantly influence the rheological behavior and curing performance of the prepared ceramic slurry. As shown in Figure 3a, the viscosity of the Si_3_N_4_ slurry follows a trend similar to that of the monomers themselves: adding high-viscosity PPTTA increased the slurry’s viscosity, while very low-viscosity HEA decreased it. It is important to note that the final slurry viscosity is also influenced by the interaction between the monomers and the surface functional groups of the ceramic particles [45]. In our study, the highly dilutable HDDA ensured that all three systems met the viscosity requirements for being evenly spread by the scraper, typically less than 5 Pa·s at a shear rate of 30 s^−1^. By adjusting the exposure energy, the curing depth of different slurries was evaluated, as depicted in Figure 3b. The curing depth of S2 and S3, which contained PPTTA, were significantly higher than that of S1. This is attributed to PPTTA having four polymerizable double bonds, which enhance the average functionality of the resin and thereby improve the curing rate [46]. It has been demonstrated that curing depth affects both dimensional accuracy and component quality. Therefore, selecting an appropriate curing depth, rather than an excessively high one, is essential (typically two to three times the layer thickness) [47]. With a layer thickness of 20 μm, the curing depths of the three pastes were controlled within the range of 40–50 μm by adjusting the exposure parameters, enabling all three slurries to achieve printing.

### 3.2. Thermogravimetric Analysis of Green Body

**Figure 4:**
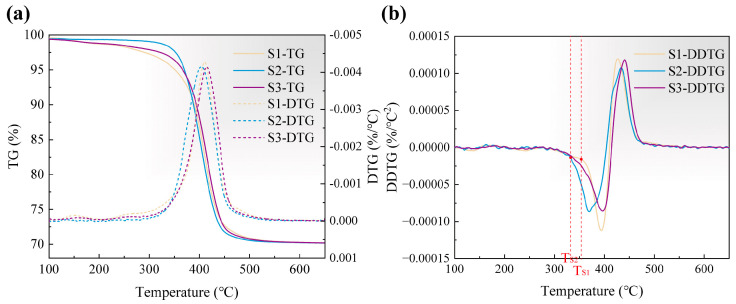
Thermal analysis of Si_3_N_4_ green bodies: (**a**) TG and DTG curves, (**b**) DDTG curves.

The kinetic analysis of resin pyrolysis is based on thermogravimetric studies of green bodies, which can reveal the reaction rates at different stages and potential transitions in kinetic processes. The TG curves of three green bodies were measured at a constant heating rate of 5 °C/min, and their first-order differential thermogravimetric (DTG) curves were derived, as illustrated in Figure 4a. The minor weight loss below 100 °C was disregarded as it primarily corresponds to the evaporation of absorbed water. The weight loss of the green bodies can be broadly categorized into two stages: a gradual stage below 300 °C and a rapid stage between 300 °C and 500 °C, with the latter exhibiting a prominent peak accompanied by a distinct shoulder in the DTG curve. This suggests that at least three pseudo-components may be assumed for further investigation. Notably, the TG curve of S1 exhibits a more gradual change, indicating that resin pyrolysis proceeds over a broader temperature range. Conversely, the steepest TG curve observed for S3 implies a concentrated release of pyrolysis gases, which could increase the risk of defect formation.

The second-order differential thermogravimetric (DDTG) curves are presented in Figure 4b. DDTG characterizes the intrinsic power of the weight loss reaction, and its pronounced turn signifies the transition in the pyrolysis mechanism [48]. Both S1 and S2 exhibit clear inflection points (T_S1_ and T_S2_), after which their DDTG curves exhibit exponential changes, reflecting a sudden increase in the intrinsic power of pyrolysis. This phenomenon is likely attributed to the rupture of the primary cross-linked network. In contrast, S3 lacks such an inflection point due to the diversity of its constituent monomers. The sequential breakage of various chain structures under thermal variation mitigates this abrupt effect.

### 3.3. 3-DAEM Kinetics Analysis of Pyrolysis

**Figure 5:**
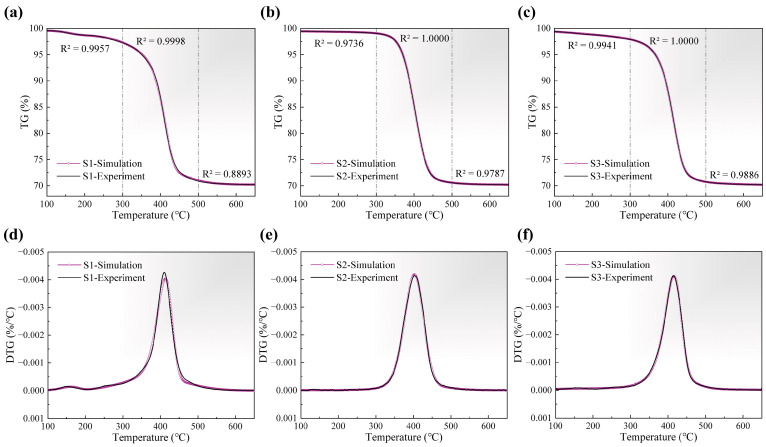
Simulation results of 3-DAEM: (**a**–**c**) comparison of TG fitting curves with experimental data and quality of piecewise fitting, (**d**–**f**) comparison of DTG fitting curves with experimental data.

Based on the prior thermogravimetric analysis, three pseudo-components were introduced to perform kinetics analysis of the pyrolysis for the three green bodies. In this study, the mean activation energy values of each pseudo-component were constrained within the range of 0–300 kJ/mol, while the standard deviation values were limited to 0–150 kJ/mol. The frequency factors were sequentially fixed at 8, 17, and 19 (common logarithm), which referred to the global fitting result. The fitting results of 3-DAEM for the TG curves are presented in Figure 5a–c. The global R^2^ values for all samples exceeded 0.9999, demonstrating an excellent agreement with the experimental data. Specifically, within the temperature range of 300–500 °C, where the most rapid weight loss occurred, the R^2^ values for S1, S2, and S3 also surpassed 0.9998. Furthermore, the comparison between the experimental and simulated DTG curves in Figure 5d–f reveals that the model can accurately capture the gradient changes, thereby validating the feasibility of using 3-DAEM to describe the kinetics of resin pyrolysis in this study. Since the vast majority of ceramics remain unchanged at the temperature of resin pyrolysis and the prior thermogravimetric analysis accounted for and reduced the heat transfer effects, variations in ceramic composition are not expected to influence the fitting performance of the model.

**Figure 6:**
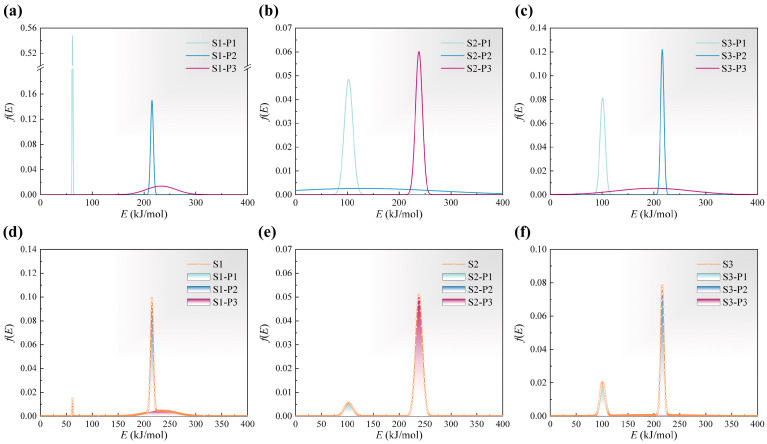
The activation energy distribution of each pseudo-component and overall activation energy distribution: (**a**,**d**) S1, (**b**,**e**) S2, (**c**,**f**) S3.

The kinetic parameters and content of each pseudo-component are summarized in Table 3. Figure 6a–c depict the activation energy distribution of each pseudo-component within the green bodies, while Figure 6d–f illustrate the overall activation energy distribution considering the relative contributions of the pseudo-components. It is worth noting that the pseudo-components within each sample are differentiated by their pre-exponential factors, which reflect distinct reaction mechanisms. Due to the complexity of the resin cross-linked network, these mechanisms are not completely the same across different samples. The apparent activation energy of each sample exhibits a bimodal distribution, with the second peak corresponding to the decomposition of the cross-linked network. Higher activation energies indicate greater energy inputs required for the reactions. The average activation energies of the main pseudo-components in S1, S2, and S3 are 215.32 kJ/mol, 238.28 kJ/mol, and 216.26 kJ/mol, respectively. This trend arises because the monofunctional monomer HEA can only form linear molecules, and its incorporation reduces the crosslinking density of polymers formed by multifunctional monomers HDDA and PPTTA, leading to the order S2 > S3 > S1. Furthermore, the frequency factor of the main pseudo-component is 17 for S1 and S3, while it is 19 for S2. Given that the model assumes parallel first-order reactions, the classical Arrhenius equation indicates that lower activation energies and frequency factors result in reduced reaction rates, lower reaction start temperatures, and slower increase in reaction rates with temperature. Finally, the integral approximation employed in the model was verified. Based on the research of Órfão et al., the larger the value of x=E/RT, the more accurate the approximation [49]. In this study, the minimum value of $x_{min}$ within the range of $E_{0,i}\pm {5\sigma }_{i}$ is 18.84. At this point, the relative deviation of the activation energy obtained using the approximation is less than 5 × 10^−5^%, and the proportion of the activation energy distribution below $x_{min}$ was extremely low.

**Figure 7:**
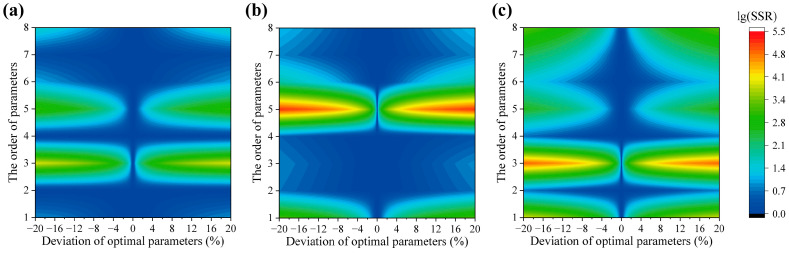
The sensitivity analysis of parameters in 3-DAEM: (**a**) S1, (**b**) S2, (**c**) S3.

Local sensitivity analysis was conducted to evaluate the influence of different parameters on the model fitting performance. In this study, a total of eight independent parameters were considered, numbered 1 to 8: $E_{0,1}$, $\sigma_{1}$, $E_{0,2}$, $\sigma_{2}$, $E_{0,3}$, $\sigma_{3}$, $c_{1},$ and $c_{2}$. Their effects were assessed within ±20% of optimized values and visualized using lg(SSR), as shown in Figure 7. Specifically, by altering one optimized parameter while keeping the others at their original optimized values, the current parameter set was obtained. SSR represents the ratio of the sum of squared residuals between the fitted values and experimental values under the current parameters to that under the optimized parameters. For each sample, parameters of the pseudo-component having a higher proportion exerted a greater influence on the variation of the model. Furthermore, among these parameters, the mean activation energy $E_{0,i}$ exhibited higher sensitivity compared to the standard deviation $\sigma_{i}$. Due to P3 accounting for 0.85 of S2, the model demonstrated very low tolerance to changes in $E_{0,3}$. In contrast, in S3, where all pseudo-components contributed significantly, the variation range was broadened.

### 3.4. Gas Pressure in Green Body

**Figure 8:**
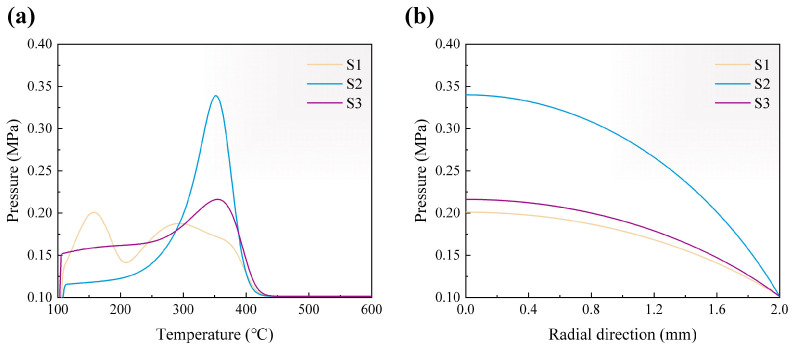
The gas pressure in green bodies: (**a**) gas pressure at the center of green bodies as temperature increases, (**b**) gas pressure in radial direction at the moment of maximum pressure.

Figure 8a illustrates the variation in gas pressure at the center of green bodies as temperature increases. The gas generated during the pyrolysis cannot be promptly expelled, leading to accumulation and the rise in pressure, which may induce cracking of the green body. The pressure peaks observed in S2 and S3 are 0.34 MPa and 0.22 MPa, occurring at 352 °C and 355 °C, respectively. The temperatures corresponding to these pressure peaks do not align with the pyrolysis rate peaks shown in the DTG curve. Instead, they occur earlier. This discrepancy arises by following mechanism. During the initial rise in pyrolysis rate, pore connectivity is limited, restricting gas release and resulting in pressure buildup. By the time the pyrolysis rate reaches its peak, sufficient gas transport pathways have formed, allowing for efficient gas discharge. This mechanism also explains why the pressure peak and curve in S2 is significantly higher and flatter compared to those in S3, as S3 undergoes a slower initial pyrolysis process. In the case of S1, the maximum pressure of 0.20 MPa occurs at 158 °C, corresponding to the first peak in the DTG curve. The rapid initial pyrolysis rate leads to a bimodal pressure distribution and an earlier shift of the pressure peak. Figure 8b presents the spatial distribution of gas pressure in green bodies at the moment of maximum pressure. The pressure decreases gradually from the center toward the edge. This model is designed to elucidate the influence of binder type with different pyrolysis kinetics on the risk of green body cracking. For more precise prediction of defect locations, additional factors must be considered, including the spatial non-uniformity of temperature and concentration fields induced by heat transfer, volume shrinkage, and evolution in the mechanical properties of the green body. Furthermore, more TG data at various heating rate are needed to decorrelate the parameters and enable kinetics models to predict the weight loss at different temperatures more accurately.

### 3.5. Debinding and Sintering

**Figure 9:**
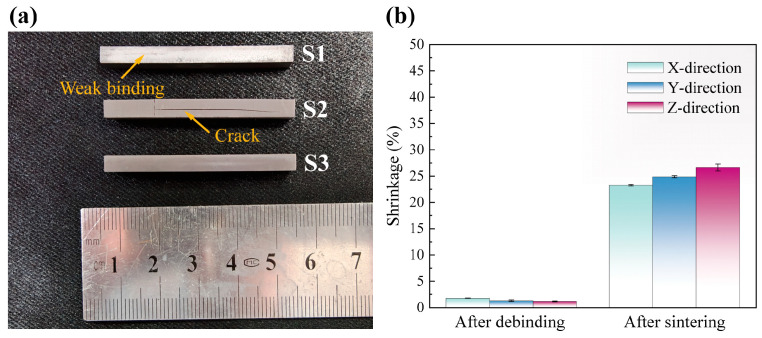
(**a**) Macroscopic morphology of the samples after debinding, (**b**) shrinkage after debinding and sintering.

The green bodies printed with three different slurries were debound using a uniform strategy, with a heating rate below 0.5 °C/min and a maximum temperature of 650 °C. The appearance of the brown bodies after debinding is shown in Figure 9a. S2 exhibited destructive cracking, which was attributed to the rapid and abrupt pyrolysis reaction, that generated a large number of gaseous products. Insufficient pyrolysis in the early stage failed to create sufficient continuous channels for gas escape, leading to excessive internal stress and subsequent cracking [20]. Additionally, weak bonding originating from the printing process was observed in some samples of S1. It has been reported that this kind of defect cannot be repaired during the subsequent sintering process and will persist in the final as-sintered body, leading to deteriorated performance [47]. Although self-healing resulting from liquid-phase sintering in silicon nitride has been reported [22], initial defects still pose a detrimental effect. Owing to excellent curing performance and milder pyrolysis characteristics, no such adverse phenomena were observed in S3. As shown in Figure 9b, the shrinkage of green bodies during the debinding process are less than 2%.

**Figure 10:**
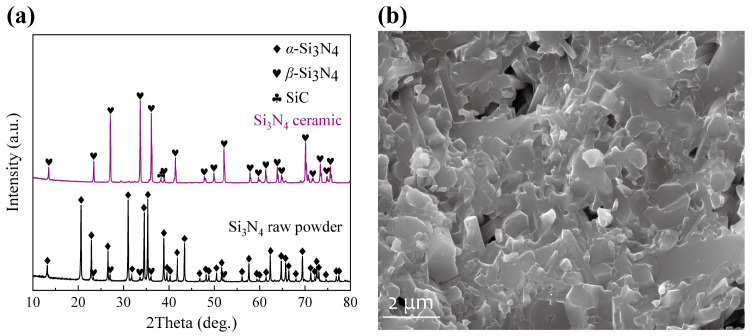
(**a**) XRD characterization of the Si_3_N_4_ raw powders and ceramics, (**b**) fracture morphology of Si_3_N_4_ ceramics.

Furthermore, Si_3_N_4_ ceramics were prepared via pressureless sintering. The sintering process is characterized by substantial dimensional shrinkage and densification. The *Z*-axis (printing direction) exhibits the most pronounced deformation, which can be attributed to the weak layer bonding. The relative density and flexural strength of sintered ceramics reach 97.16 ± 1.34% and 577.87 ± 107.29 MPa. Figure 10a presents the XRD patterns of Si_3_N_4_ raw powders and sintered ceramics. Liquid-phase sintering serves as the primary densification mechanism for Si_3_N_4_ ceramics in this study. The *α*-Si_3_N_4_ phase in raw powders undergone a dissolution-precipitation process, transforming into *β*-Si_3_N_4_. Additionally, a minor amount of SiC formed from residual carbon which remained after debinding. Y-Al-Si-N-O crystalline phases, showing some impurity peaks, precipitated from the liquid phase generated by the sintering aids during the cooling stage. Figure 10b displays the fracture morphology of the ceramic, revealing few pores and elongated grain features. The coexistence of transgranular and intergranular fracture features aligns with the typical fracture behavior of silicon nitride ceramics primarily composed of the *β* phase.

## 4. Conclusions

In summary, this study has introduced a novel perspective on the debinding process of ceramic vat photopolymerization manufacturing based on the kinetics of resin pyrolysis. The adaptability to green bodies with varying monomer compositions and sensitivity to fitting parameters of M-DAEM were evaluated. Furthermore, the M-DAEM was integrated into a one-dimensional numerical model to elucidate the effect of pyrolysis kinetics on the internal gas pressure in green bodies during debinding. Results indicate that the M-DAEM accurately describes the pyrolysis kinetics of cured resin applied in this study, achieving a global fitting quality with R^2^ values exceeding 0.9999. The activation energies of the primary pseudo-components in S1, S2, and S3 were calculated to be 215 kJ/mol, 238 kJ/mol, and 216 kJ/mol, respectively. The sensitivity analysis revealed that deviations in the parameters of pseudo-components with higher proportions and mean activation energy significantly influenced the variation of the model. Numerical analysis corroborated by TG and experimental results demonstrated that incorporating the monofunctional monomer HEA facilitates pyrolysis at lower temperatures and rates, suppresses the peak value of gas pressure, and then effectively suppresses defect formation during the debinding process. Multifunctional monomers enhance the curing depth of slurries, contributing to more stable printing outcomes. This study provides a valuable approach for optimizing binders in ceramic VPP.

## Figures and Tables

**Table 1:** Components of the Si_3_N_4_ ceramic slurry.

Samples	HEA ^a^	HDDA ^a^	PPTTA ^a^	Dispersant ^b^ wt.%	Photoinitiator ^a^ wt.%	Ceramics ^c^ vol.%
	vol.%					
S1	60	40	/	3	1	40
S2	/	40	60			
S3	30	40	30			

a: Relative to the monomer mixture, b: Relative to the ceramic powders, c: Relative to the ceramic slurry.

**Table 2:** Values of the parameters used in the simulations.

Symbol and Description	Value and Units
$T_{ini}$ initial temperature	373.15 K
$T_{fin}$ final temperature	873.15 K
β heating rate	5 K/min
$r_{0}$ radius of green body	2 mm
$V_{c}$ volume fraction of ceramic particles	0.40
$V_{0}$ volume fraction of initial pores	0.05
d diameter of ceramic particles	0.7 μm
$M_{g}$ molecular mass of pyrolysis gas	0.086089 kg/mol
$\rho_{p}$ the density of cured resin	S1 1069.88 kg/m^3^
	S2 1094.60 kg/m^3^
	S3 1082.24 kg/m^3^

**Table 3:** The kinetic parameters and content of pseudo-components.

Samples	Pseudo-Components	$E_{0,i}$ (kJ/mol)	$\sigma_{i}$ (kJ/mol)	$\log_{10}A_{i}$	$c_{i}$
S1	P1	62.11	0.73	8	0.03
	P2	215.32	2.66	17	0.64
	P3	233.12	29.03	19	0.34
S2	P1	102.45	8.26	8	0.11
	P2	128.12	150.14	17	0.04
	P3	238.28	6.63	19	0.85
S3	P1	101.08	4.90	8	0.24
	P2	216.26	3.27	17	0.64
	P3	199.43	72.94	19	0.12

## Data Availability

The original contributions presented in this study are included in the article. Further inquiries can be directed to the corresponding authors.

## References

[1] Zheng W., Wu J.-M., Chen S., Wang C.-S., Liu C.-L., Hua S.-B., Yu K.-B., Zhang J., Zhang J.-X., Shi Y.-S. (2021). Influence of Al_2_O_3_ content on mechanical properties of silica-based ceramic cores prepared by stereolithography. J. Adv. Ceram..

[2] Li F.-F., Ma N.-N., Chen J., Zhu M., Chen W.-H., Huang C.-C., Huang Z.-R. (2022). SiC ceramic mirror fabricated by additive manufacturing with material extrusion and laser cladding. Addit. Manuf..

[3] Solórzano-Requejo W., Martínez Cendrero A., Altun A.A., Nohut S., Ojeda C., García Molleja J., Molina-Aldareguia J., Schwentenwein M., Díaz Lantada A. (2024). Topology optimisation and lithography-based ceramic manufacturing of short-stem hip prostheses with enhanced biomechanical and mechanobiological performance. Virtual Phys. Prototyp..

[4] Montazerian M., Baino F., Fiume E., Migneco C., Alaghmandfard A., Sedighi O., DeCeanne A.V., Wilkinson C.J., Mauro J.C. (2023). Glass-ceramics in dentistry: Fundamentals, technologies, experimental techniques, applications, and open issues. Prog. Mater. Sci..

[5] Kostretsova N., Pesce A., Hofmann C., Neuberg S., Babeli I., Nuñez M., Morata A., Kolb G., Torrell M., Tarancón A. (2025). Enhanced CO_2_ methanation with ceramic 3d printed catalyst bed reactor. Chem. Eng. J..

[6] Gao C., Li X., Xu W., Chen Y., Luo T., Gao R., Cui J., Chu X., Wen X., Zhou W. (2025). 3D printing of carbon/ceramic conductive composites as joule-heating catalyst support for hydrogen production. Addit. Manuf..

[7] Liu G., Zhang X., Chen X., He Y., Cheng L., Huo M., Yin J., Hao F., Chen S., Wang P. (2021). Additive manufacturing of structural materials. Mater. Sci. Eng. R Rep..

[8] Rasaki S.A., Xiong D., Xiong S., Su F., Idrees M., Chen Z. (2021). Photopolymerization-based additive manufacturing of ceramics: A systematic review. J. Adv. Ceram..

[9] Miao W.-J., Wang S.-Q., Wang Z.-H., Wu F.-B., Zhang Y.-Z., Ouyang J.-H., Wang Y.-M., Zou Y.-C. (2025). Additive manufacturing of advanced structural ceramics for tribological applications: Principles, techniques, microstructure and properties. Lubricants.

[10] Kanehira S., Kirihara S., Miyamoto Y. (2005). Fabrication of TiO_2_–SiO_2_ photonic crystals with diamond structure. J. Am. Ceram. Soc..

[11] Li B., Li Z., Cooperstein I., Shan W., Wang S., Jiang B., Zhang L., Magdassi S., He J. (2023). Additive manufacturing of transparent multi-component nanoporous glasses. Adv. Sci..

[12] Zhao J.-J., Zhang Y.-Z., Li J.-H., Wang Z.-H., Miao W.-J., Wu F.-B., Wang S.-Q., Ouyang J.-H. (2025). Additive manufacturing of alumina-based ceramic structures by vat photopolymerization: A review of strategies for improving shaping accuracy and properties. Materials.

[13] He R., Liu W., Wu Z., An D., Huang M., Wu H., Jiang Q., Ji X., Wu S., Xie Z. (2018). Fabrication of complex-shaped zirconia ceramic parts via a dlp-stereolithography-based 3d printing method. Ceram. Int..

[14] Wang Z.H., Zhang Y.Z., Miao W.J., Wu F.B., Wang S.Q., Ouyang J.H., Wang Y.M., Zou Y.C. (2025). Vat photopolymerization-based additive manufacturing of Si_3_N_4_ ceramic structures: Printing optimization, debinding/sintering, and applications. Materials.

[15] Sheng P., Nie G., Li Y., Wang L., Chen J., Deng X., Wu S. (2023). Enhanced Curing Behavior, Mechanical and thermal properties of 3d printed aluminum nitride ceramics using a powder coating strategy. Addit. Manuf..

[16] Qu P., Liang G., Hussain M.I., Hanif M., Hamza M., Huang K., Lou Y., Chen Z. (2025). Low-temperature fabrication of high-specific strength SiC-based ceramics via photopolymerization 3d printing with controllable anisotropy. Int. J. Extrem. Manuf..

[17] Eckel Z.C., Zhou C., Martin J.H., Jacobsen A.J., Carter W.B., Schaedler T.A. (2016). Additive manufacturing of polymer-derived ceramics. Science.

[18] Yu X., Zhao Y., Wang Z., Wang Y., Yu Z., Zhong K., Zhao J. (2024). Microstructure formation mechanisms and property regulation methods during ceramic additive manufacturing. J. Manuf. Process..

[19] Ma W., Zheng K., Quan Y., Lian Q., Zhuang J., Qi C., Qi S., Zhang J., Li H., Liu W. (2024). High-precision complex structured Sm-PMN-PT ceramics with large piezoelectric response manufactured by vat photopolymerization. Addit. Manuf..

[20] Zhou S., Liu G., Wang C., Zhang Y., Yan C., Shi Y. (2024). Thermal debinding for stereolithography additive manufacturing of advanced ceramic parts: A comprehensive review. Mater. Des..

[21] Gu Q., Wang H., Gao W., Yu J., Zhou X. (2023). Preparation of large-size alumina ceramic parts by dlp 3d printing using high-solid-loading paste and optimizing the debinding process. Ceram. Int..

[22] Marie T., Du Z., Gan C.L., Marinel S., Sridharan V.S., Manière C. (2025). Debinding and sintering optimization of stereolithography based silicon nitride parts for attaining centimetric wall-thickness shapes. J. Eur. Ceram. Soc..

[23] Li J., Zhang C., Yin R., Zhang W. (2019). DAEM kinetics analysis and finite element simulation of thermal debinding process for a gelcast SiAlON green body. Ceram. Int..

[24] Zhao K., Ye Z., Su Z., Cao W., Shi D., Hao X., Zhang S., Wang Z., Xu X., Zhu J. (2025). A diffusion-controlled kinetic model for binder burnout in a green part fabricated by binder jetting based on the thermal decomposition kinetics of TEG-DMA. Addit. Manuf..

[25] Kumar A., Gokhale A., Ghosh S., Aravindan S. (2019). Effect of nano-sized sintering additives on microstructure and mechanical properties of Si_3_N_4_ ceramics. Mater. Sci. Eng. A.

[26] Li Y., Huang S., Wang S., Zhang X., Wang Y., Lu B., Luo Y., He F., Liu W., Wu S. (2022). Research on the effects of surface modification of ceramic powder on cure performance during digital light processing (dlp). Ceram. Int..

[27] Drobecq I., Bigot C., Soppera O., Malaquin L., Venzac B. (2025). Optimizing dimensional accuracy in two-photon polymerization: Influence of energy dose and proximity effects on sub-micrometric fiber structures. Addit. Manuf..

[28] (2016). Fine Ceramics (Advanced Ceramics, Advanced Technical Ceramics)—Test Method for Flexural Strength of Monolithic Ceramics at Room Temperature.

[29] Zou J., Hu H., Rahman M.M., Yellezuome D., He F., Zhang X., Cai J. (2022). Non-isothermal pyrolysis of xylan, cellulose and lignin: A hybrid simulated annealing algorithm and pattern search method to regulate distributed activation energies. Ind. Crops Prod..

[30] Chen Z., Hu M., Zhu X., Guo D., Liu S., Hu Z., Xiao B., Wang J., Laghari M. (2015). Characteristics and kinetic study on pyrolysis of five lignocellulosic biomass via thermogravimetric analysis. Bioresour. Technol..

[31] Vyazovkin S., Burnham A.K., Criado J.M., Pérez-Maqueda L.A., Popescu C., Sbirrazzuoli N. (2011). ICTAC kinetics committee recommendations for performing kinetic computations on thermal analysis data. Thermochim. Acta.

[32] Senum G.I., Yang R.T. (1977). Rational approximations of the integral of the arrhenius function. J. Therm. Anal..

[33] Koga N. (1994). A review of the mutual dependence of arrhenius parameters evaluated by the thermoanalytical study of solid-state reactions: The kinetic compensation effect. Thermochim. Acta.

[34] Das P., Tiwari P. (2017). Thermal degradation kinetics of plastics and model selection. Thermochim. Acta.

[35] Várhegyi G., Bobály B., Jakab E., Chen H. (2010). Thermogravimetric study of biomass pyrolysis kinetics: A distributed activation energy model with prediction tests. Energy Fuels.

[36] Jain A.A., Mehra A., Ranade V.V. (2016). Processing of TGA data: Analysis of isoconversional and model fitting methods. Fuel.

[37] Lin Y., Chen Z., Dai M., Fang S., Liao Y., Yu Z., Ma X. (2018). Co-pyrolysis kinetics of sewage sludge and bagasse using multiple normal distributed activation energy model (M-DAEM). Bioresour. Technol..

[38] Tsai D.S. (2004). Pressure buildup and internal stresses during binder burnout: Numerical analysis. AIChE J..

[39] Feng K., Lombardo S.J. (2004). Modeling of the pressure distribution in three-dimensional porous green bodies during binder removal. J. Am. Ceram. Soc..

[40] Tseng C.-C., Li K.-L., Chao J.-H. (2025). Numerical investigations of the binder burnout process during the MLCC manufacturing. Appl. Therm. Eng..

[41] Doménech-Carbó M.T., Bitossi G., Osete-Cortina L., Yusá-Marco D.J. (2009). Study of ageing of ketone resins used as picture varnishes by pyrolysis–silylation–gas chromatography–mass spectrometry. J. Anal. Appl. Pyrolysis.

[42] Matsubara H., Yoshida A., Kondo Y., Tsuge S., Ohtani H. (2003). Characterization of network structures in UV-cured acrylic ester resin by pyrolysis−gas chromatography in the presence of organic alkali. Macromolecules.

[43] Matsubara H., Yoshida A., Ohtani H., Tsuge S. (2002). Compositional analysis of UV-cured acrylic ester resins by pyrolysis–gas chromatography in the presence of organic alkali. J. Anal. Appl. Pyrolysis.

[44] Gallant R.W. (1968). Physical Properties of Hydrocarbons.

[45] Li K., Zhao Z. (2017). The effect of the surfactants on the formulation of UV-curable SLA alumina suspension. Ceram. Int..

[46] Oezkan B., Sameni F., Karmel S., Engstrøm D.S., Sabet E. (2021). A systematic study of vat-polymerization binders with potential use in the ceramic suspension 3d printing. Addit. Manuf..

[47] Li X., Su H., Dong D., Jiang H., Liu Y., Shen Z., Guo Y., Zhang Z., Guo M. (2024). Selection strategy of curing depth for vat photopolymerization 3d printing of Al_2_O_3_ ceramics. Addit. Manuf..

[48] Zhang Y., Zhang Y., Li Y., Shi X., Che B. (2022). Determination of ignition temperature and kinetics and thermodynamics analysis of high-volatile coal based on differential derivative thermogravimetry. Energy.

[49] Órfão J.J.M. (2007). Review and evaluation of the approximations to the temperature integral. AIChE J..

